# Broadband impedance modulation via non-local acoustic metamaterials

**DOI:** 10.1093/nsr/nwab171

**Published:** 2021-09-11

**Authors:** Zhiling Zhou, Sibo Huang, Dongting Li, Jie Zhu, Yong Li

**Affiliations:** Institute of Acoustics, School of Physics Science and Engineering, Tongji University, Shanghai 200092, China; Institute of Acoustics, School of Physics Science and Engineering, Tongji University, Shanghai 200092, China; Department of Mechanical Engineering, The Hong Kong Polytechnic University, Hong Kong, China; Institute of Acoustics, School of Physics Science and Engineering, Tongji University, Shanghai 200092, China; Department of Mechanical Engineering, The Hong Kong Polytechnic University, Hong Kong, China; The Hong Kong Polytechnic University Shenzhen Research Institute, Shenzhen 518057, China; Institute of Acoustics, School of Physics Science and Engineering, Tongji University, Shanghai 200092, China

**Keywords:** impedance modulation, acoustic absorption, non-local metamaterials

## Abstract

Causality of linear time-invariant systems inherently defines the wave-matter interaction process in wave physics. This principle imposes strict constraints on the interfacial response of materials on various physical platforms. A typical consequence is that a delicate balance has to be struck between the conflicting bandwidth and geometric thickness when constructing a medium with desired impedance, which makes it challenging to realize broadband impedance modulation with compact structures. In pursuit of improvement, the over-damped recipe and the reduced excessive response recipe are creatively presented in this work. As a proof-of-concept demonstration, we construct a metamaterial with intensive mode density that supports strong non-locality over a frequency band from 320 Hz to 6400 Hz. Under the guidelines of the over-damped recipe and the reduced excessive response recipe, the metamaterial realizes impedance matching to air and exhibits broadband near-perfect absorption without evident impedance oscillation and absorption dips in the working frequency band. We further present a dual-functional design capable of frequency-selective absorption and reflection by concentrating the resonance modes in three frequency bands. Our research reveals the significance of over-damped recipe and the strong non-local effect in broadband impedance modulation, which may open up avenues for constructing efficient artificial impedance boundaries for energy absorption and other wave manipulation.

## Teaser

The nonlocality between subunits was utilized to approach the causality-governed minimal thickness, thereby enabling ultrathin acoustic metamaterials to achieve broadband sound absorption and desired impedance profiles over four octaves.

## INTRODUCTION

Causality universally governs the evolution of linear time-invariant wave systems. It inherently restricts the modulations that different materials can provide to propagating waves at interfaces. This results in the causality constraint, delineated by the minimal structural thickness for a designated bandwidth of impedance modulation. For centuries, broadband impedance modulation has been investigated in various classical wave systems. In particular, modulations for broadband impedance matching have long been pursued owing to the scientific and engineering values in wave physics. For airborne sound, two types of conventional solutions have been proposed to provide impedance matching. The first type is ‘globally reacting’ materials composed of microstructures, such as porous and fibrous materials [1], which can be macroscopically regarded as homogeneous materials with wave energy dissipated during propagation. However, the thicknesses of these materials need to be comparable to the maximal working wavelength. To reduce the thickness, locally resonant structures such as microperforated panel resonators are proposed [2], which can enhance energy density in the low-frequency regime for better impedance matching. However, for these locally resonant structures, the inevitable strong dispersion constrains the impedance modulation to a narrow bandwidth.

The recent emergence of acoustic metamaterials [3–17] provides fascinating opportunities for wave manipulation. In particular, metamaterial-based devices with ultra-thin thickness can construct artificial boundaries unattainable in natural materials, leading to numerous applications in impedance and wavefront engineering [8,9,18,19]. In pursuit of impedance matching boundaries with deep subwavelength thickness, several acoustic metamaterials have been presented, including decorated membranes [20,21], coiled channels [22–24] and specially designed Helmholtz resonators (HRs) [25–28]. However, these structures feature local resonance, which results in strong dispersion and therefore largely limits the working bandwidth. To achieve broadband impedance matching, the approach of coupling local resonances is presented. Typically, coupled metamaterials via curled Fabry-Pérot (FP) channels [29–32] or HRs [33–35] can effectively broaden the operating bandwidth. Nevertheless, the antiresonances induced by the unexpected interactions of the local resonances cause rapid impedance oscillation and result in absorption dips with the bandwidth broadening. Recently, studies on non-local metamaterials have drawn increasing attention [36–41]. Strong non-local coupling has been proven to enable extreme acoustic anisotropy [36], reduce higher orders of diffractions [37], generate anomalous reflection [38], improve sound-absorption performance [42] and improve other wave-manipulation performances [39–41]. So far, rare research achieves broadband acoustic impedance modulation against dispersion by utilizing non-locality. But actually, the non-locality induced by lateral energy channeling in the near field has great potential to suppress dispersion and empower superior impedance modulation.

In this work, we present the concept of utilizing strong non-locality to achieve efficient broadband impedance modulation. Distinct from the works based on a connective bridge structure among the subunits [36,42], the non-locality is induced by radiative coupling in the near field supported by an intensive mode density. Besides, we propose the intrinsically over-damped recipe and the reduced excessive response recipe to achieve the minimal thickness governed by the causality principle. For demonstration, the cascade neck-embedded Helmholtz resonators that can support more resonance modes are employed to construct a non-local metamaterial consisting of 36 subunits with a thickness of 100 mm. We demonstrate that the intensive mode density of the metamaterial supports strong non-local coupling among specific subunits, which can significantly suppress the impedance oscillations and absorption dips at antiresonance frequencies. As a result, the desired impedance profile from 320 Hz to 6400 Hz is achieved, leading to broadband quasi-perfect sound absorption without evident absorption dips. In addition, we realize dual-function impedance modulation by concentrating the modes in three frequency bands. In the mode-concentrated bands, stronger non-local coupling is induced and the metamaterial acts as an absorbing boundary, while in the remaining bands the metamaterial serves as a reflective boundary.

## RESULTS AND DISCUSSION

### Causality constraint and acoustic impedance modulation

Acoustic impedance describes how the material reacts to external excitation and is defined as *Z*_a_ = *p*/*V*, where *p* and *V* denote the sound pressure and volume velocity respectively. Specifically, the absorption coefficient of acoustic material under normal incidence can be calculated with the impedance according to

(1) \begin{eqnarray*} A(\omega ) = 1 - {| {r(\omega )} |^2} = 1 - {\left| {\frac{{{Z_{\rm {a}}}(\omega ) - {Z_{\rm {0}}}/S}}{{{Z_{\rm {a}}}(\omega ) + {Z_{\rm {0}}}/S}}} \right|^2},\nonumber\\ \end{eqnarray*}

where ω is the angular frequency; *A*(ω) and *r*(ω) denote the frequency-dependent absorption and reflection coefficients, respectively; *Z*_0_ is the characteristic impedance of the surrounding medium; and *S* is the area of the incident field. The impedance-matching condition is defined as *Z*_a_ = *Z*_0_/*S*, which ensures total absorption (*A* = 1) of the incident sound energy.

**Figure 1.**
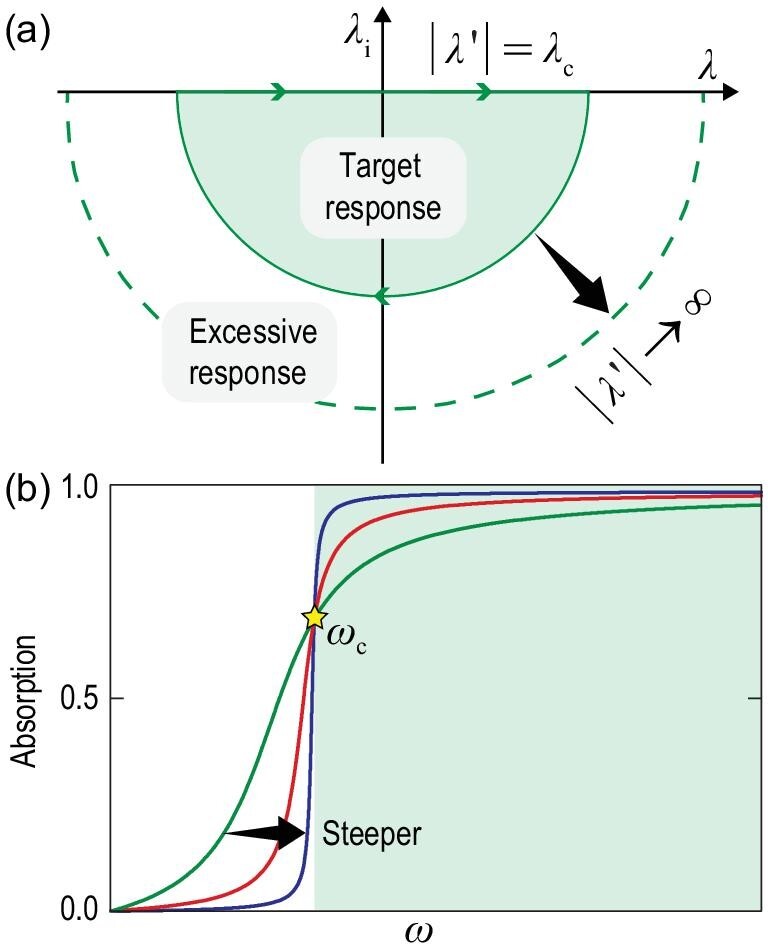
(a) Illustration of the frequency response of acoustic materials in the complex wavelength plane. Target response and excessive response are associated with the absorption-dominated and reflection-dominated spectra in (b), respectively. The semicircle with a radius of |λ| denotes the contour of integral in the causality constraint. The causality principle is established at the static limit (|λ| → ∞) including the excessive response. (b) Absorption spectra relating to different response functions. The green, red and blue curves demonstrate the increasing slope responses with unanimous *L*_min_ of 100 mm. A greater slope indicates better absorption performance above the critical frequency ω_c_.

In principle, the modulation of acoustic impedance is restricted by the causal nature of linear time-invariant systems. This can be described by *A*(λ) on the complex wavelength plane [43] with λ^′^ = λ + j(λ_e_ + λ_i_), where λ is the real wavelength, λ_e_ denotes the intrinsic dissipation and λ_i_ denotes the additional dissipation. Here, the propagation factor is e^j2π*x*/λ^. On the real axis where λ_i_ = 0, *A*(λ) represents the absorption when the system is excited by an incident wave with a wavelength of λ. Meanwhile, different λ and λ_i_ on the lower-half plane depict the intrinsic loss of the system. Therefore, the real axis and the lower-half plane denote two manifestations of the interaction between the internal system and external excitation. The dynamics of this process can be described by a path consisting of a straight line on the real axis and a semi-circle with an infinite radius on the lower-half plane [Fig. 1(a)]. As a result of the causal nature, *A*(λ) can be solved reversely by accumulating the dissipation on the lower-half plane, leading to an inequality constraint on the geometric thickness *L*, which is written as [31,43]

(2) \begin{eqnarray*} L &\ge& \frac{{\rm {1}}}{{2{\pi ^2}\phi }}\bigg | {\int _0^\infty {{\rm {ln}}\bigg | {\frac{{{Z_{\rm {a}}}(\lambda ) - {\rho _0}{c_0}/S}}{{{Z_{\rm {a}}}(\lambda ) + {\rho _0}{c_0}/S}}} \bigg |{\rm {d}}\lambda } } \bigg | \nonumber\\ &=& \frac{{\rm {1}}}{{4{\pi ^2}\phi }}\bigg | {\int _0^\infty {{\rm {ln}}| {1 - A(\lambda )} |{\rm {d}}\lambda } } \bigg | = {L_{\min }},\nonumber\\ \end{eqnarray*}

where φ = *V*_air_/*V*_0_ is the volume fraction of the material; ρ_0_ = 1.21 kg/m^3^ and *c*_0_ = 343 m/s are the static density and speed of sound in air at 20 °C; and *L*_min_ is the minimal thickness. Equation (2) demonstrates that the thickness of the material is quantitatively subject to its frequency response manifested by *Z*(λ) or *A*(λ), which may possess equivalent significance as the quality factor of the HR (see Note 1 within the online supplementary material). For certain *Z*(λ) or *A*(λ), the material is defined to be optimal when *L* = *L*_min_ is approximately attained. Otherwise, it is non-optimal and the same *Z*(λ) or *A*(λ) can be realized with a smaller thickness *L* (see Note 1 within the online supplementary material for details).

To realize broadband impedance matching along with near-perfect absorption starting from ω_c_ via a structure reaching the minimal thickness, the response feature of the sound-absorbing system is explored in the following sections. Assume that the absorption spectrum *A*(ω) can be manipulated arbitrarily with the same *L*_min_, then some interesting conclusions can be drawn. We investigate three hypothetical response functions *A*(ω) (see Note 2 within the online supplementary material) with the same *L*_min_ = 100 mm and φ = 0.7 [Fig. 1(b)]. The comparison shows that, when absorption under ω_c_ is weaker, a better absorption performance can be realized in the target band. Based on this revelation, the efficiency of the impedance-matching boundary can be improved by suppressing the excessive response under ω_c_ and intuitively realizing a steeper curve. Actually, the response schematic in Fig. 1(a) fundamentally explains this feature. The green area surrounded by a radius of λ_c_ contributes to the target absorption spectrum above ω_c_, so we define it as the target response. The area outside the target response (the white region) is associated with the absorption spectrum under ω_c_ that has a small contribution to the overall absorption performance. But it still has comparatively high weight in *L*_min_ because of the large magnitude of the logarithmic function when λ → ∞ [Eq. (2)]. For this reason, we define it as the excessive response. This is an essential feature of absorption. For example, porous or fibrous materials tend to comply with the smaller slope case [green line in Fig. 1(b)] with rather strong absorption under ω_c_, leading to a thickness comparable to the working wavelength. In what follows, we also demonstrate that the excessive response can be effectively suppressed via a coupled-resonant structure [red line in Fig. 1(b)], and a broadband impedance-matching boundary with deep subwavelength thickness can be achieved.

Therefore, although all optimal sound-absorbing materials with the same thickness are physically equal according to the causality principle, it is still a huge challenge to increase the slope of the absorption coefficient curve around ω_c_ and improve the absorption performance in the target band by making maximum use of the geometric thickness.

### Subunits of non-local metamaterials

**Figure 2.**
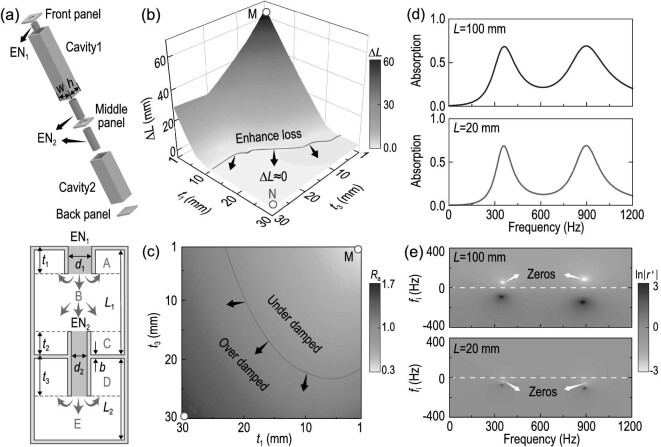
(a) Schematic of the cascade neck-embedded Helmholtz resonator (CNEHR). Here EN_1_ and EN_2_ refer to the upper and the lower embedded necks, respectively; $w$ and *h* are the width and length of the subunit including the wall thickness *b*. The green arrows illustrate the propagation paths among five domains noted by A, B, C, D and E. (b) Redundant thickness (Δ*L* = |*L* − *L*_min_|) with varying lengths (*t*_1_ and *t*_2_) of EN_1_ and EN_2_. The red curve indicates the boundary between the redundant region (Δ*L* > 0) and the optimal region (Δ*L* ≈ 0). (c) Resistance *R*_a_ at the first-order resonance frequency corresponds to the CNEHR in (b) with varying lengths *t*_1_ and *t*_2_. The red curve indicates *R*_a_ = 1. (d) Theoretical absorption spectra of two different CNEHRs with thicknesses of 100 mm and 20 mm. (e) Reflection coefficients in the complex frequency plane correspond to the structures in (d). Detailed geometric parameters are listed in Note 11 of the online supplementary material.

Further, resonant structures are employed for demonstration. The HR can provide resonance modes at specific frequencies, making it a good candidate for strong tailoring and flexible manipulation of the impedance. Based on the HR, we construct the cascade neck-embedded Helmholtz resonator (CNEHR) as the subunit of the metamaterial [Fig. 2(a)]. Compared with classic HR, the necks in the CNEHR are reversed and embedded in the cavity to realize a compact structure, namely, the embedded necks (ENs) [25]. Furthermore, to obtain more resonance modes for the suppression of antiresonances (demonstrated later), a middle panel with embedded neck EN_2_ is inserted into the structure, partitioning the entire cavity into two parts (cavity 1 and cavity 2). Benefitting from the multidegrees of freedom of geometric parameters, the CNEHR possesses extraordinary functionalities to manipulate the acoustic response [24,25,34]. To obtain the surface impedance of the CNEHR, transfer matrix theory is developed to describe the relationship of pressure and velocity fields between the bottom (*P*_b_, *V*_b_) and top (*P*_t_, *V*_t_) boundaries [44,45]:

(3) \begin{equation*} \left(\begin{array}{c}{P_{\rm {t}}}\\ {{V_{\rm {t}}}}\end{array}\right) = {\rm {T}} \left(\begin{array}{c}{P_{\rm {b}}}\\ {{V_{\rm {b}}}}\end{array}\right) . \end{equation*}

Here $\rm {T}$ is the transfer matrix of the subunit (see Note 3 within the online supplementary material for the detailed derivation). Then the surface impedance can be expressed as *Z*_a*n*_ = *P*_t_/*V*_t_. Finally, the absorption spectrum can be calculated with Eq. (1) (see Notes 3 and 4 within the online supplementary material for the experimental verification of the surface impedance model of a CNEHR).

### Over-damped nature of the optimal system

To show the physics behind Eq. (2), a redundant thickness defined as Δ*L* = |*L* − *L*_min_| is employed to illustrate the difference between the current system and the optimal one. Substituting the acoustic impedance *Z*_a*n*_ of a CNEHR into Eq. (2), the dependence of Δ*L* on parameters *t*_1_ and *t*_3_ can be illustrated [Fig. 2(b)]. Obviously, it reads Δ*L* > 0 at point M (*t*_1_ = *t*_2_ = 1 mm), revealing that the system is far from the optimal one governed by the causality principle. As *t*_1_ and *t*_2_ increase, Δ*L* reduces and eventually arrives at an optimal region where Δ*L* ≈ 0. Meanwhile, the corresponding acoustic resistance *R*_a_ is enhanced from 0.3 to 1.7 [Fig. 2(c)], which demonstrates that the damping of the CNEHR transforms from under damped to over damped [46] during this process (from point M to point N).

To further uncover the underlying mechanism, two CNEHRs with thicknesses of 100 mm and 20 mm are investigated. The theoretical absorption spectra under normal incidence are calculated and compared in Fig. 2(d) (the corresponding minimal thicknesses are 44.9 mm and 19.5 mm). It can be observed that the two CNEHRs with markedly different thicknesses exhibit nearly indistinguishable absorption spectra. However, their response characteristics are distinct on the complex frequency plane [47] where an imaginary part *f*_i_ is employed to introduce additional dissipation and reveal the damping state of the system. Similar to the complex wavelength, the complex frequency *f*^ ′^ = *f* + j(*f*_e_ + *f*_i_) can be obtained, where *f* is the real frequency and *f*_e_ denotes the intrinsic dissipation of the system. Therefore, the complex frequency responses of the two CNEHRs in Fig. 2(d) represented by the logarithm of reflection coefficient ln |*r*^′^| can be illustrated with a two-dimensional (2D) color map [Fig. 2(e)] with varying *f* and *f*_i_, where the two zeros imply the minimum of ln |*r*^′^| and reflect two resonance modes of the CNEHR. Obviously, the CNEHRs with *L* = 100 mm and *L* = 20 mm respectively show two zeros on the upper- and lower-half planes distinctly, suggesting that the corresponding intrinsic losses are insufficient and excessive. Along with Fig. 2(b) and (c), it can be concluded that the damping state of the system and zeros on the complex frequency plane are closely related to the thicknesses of the materials. Actually, this phenomenon stems from the response function *A*(λ), which leads to a deduction [43] expressed as

(4) \begin{equation*} L = {L_{\min }} - \frac{1}{{2\pi \phi }}\sum \limits _{n} {{\mathop {\rm Im}\nolimits } ({\lambda _n})}, \end{equation*}

where Im(λ_*n*_) is the imaginary part of the complex wavelength leading to zeros of ln |*r*^′^| on the lower-half complex wavelength plane (corresponding to the upper-half complex frequency plane). Hence, the underlying physical mechanism of the results demonstrated in Fig. 2 can be revealed: insufficient damping of the system leads to a flawed response featuring the presence of zeros on the upper half of the complex frequency plane, which mathematically results in a redundant term in Eq. (4) and enlarges the thickness *L*. This fundamentally explains why the damping state is crucial for realizing optimal impedance modulation and compacting the thickness of the materials.

This comparison indicates that the absorption spectrum is a simple representation, while the complex frequency plane depicts the radical response of acoustic materials. These conclusions can be extended to broadband scenarios because the causality principle is universally valid in linear time-invariant systems. Consequently, the over-damped recipe can be utilized in impedance modulation for broadband near-perfect absorption.

### Broadband impedance matching via a non-local metamaterial

**Figure 3.**
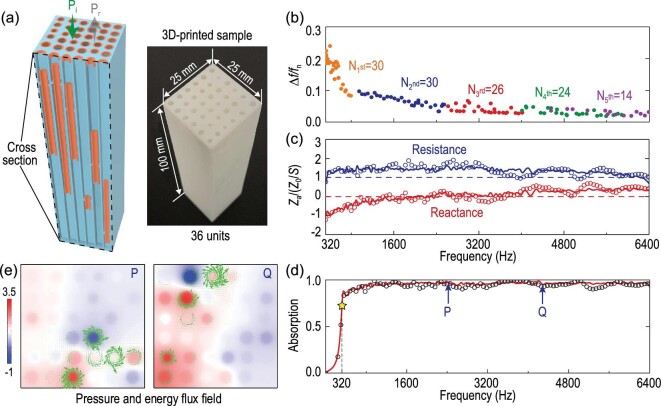
(a) Schematic of the non-local metamaterial consisting of 36 CNEHRs (the right panel shows the 3D-printed sample). The wall thickness is set at 0.5 mm. (b) Theoretical half-maximum relative bandwidth of resonance modes. The sequence of numbers 1^st^ to 5^th^ denotes the order of the resonance modes; the subsequent numbers are the numbers of resonance modes. Modes with absorption peaks lower than 0.1 are ignored in the graph. (c) Theoretical (lines) and measured (circles) acoustic resistance and reactance of the non-local metamaterial absorber. (d) Theoretical (lines) and measured (circles) absorption spectra of the non-local metamaterial absorber. Arrows P and Q respectively denote a strong absorption and a comparatively weak one. (e) Simulated pressure (indicated by color) and energy flux (indicated by green arrows) fields at 0.1 mm above the non-local metamaterial. The two graphs correspond to the two specific frequencies (2430 Hz and 4300 Hz) noted by arrows (P and Q) in (e).

From above, two essential guidelines of impedance modulation for realizing broadband near-perfect absorption can be deduced: the over-damped recipe and the reduced excessive response recipe. During the implementation process, it is found that relatively smaller cross-sectional areas of the subunits can lead to larger slopes of the absorption spectrum (see Note 5 within the online supplementary material for details). By employing more subunits, the excessive response of the system can be significantly reduced. Furthermore, the employment of more subunits can lead to an intensive mode density and support strong non-local coupling, which is theoretically demonstrated to suppress the antiresonances [48] (see Note 10 within the online supplementary material). With the guidelines above, 36 CNEHRs are employed to construct the metamaterial [Fig. 3(a)]. Because the cross-sectional dimension of the metamaterial is subwavelength in the frequency band of interest, the surface impedance of the metamaterial can be calculated from [34]

(5) \begin{equation*} {Z_{\rm {a}}} = {\bigg ( {\sum \limits _{{{n}} = 1}^{{N}} {Z_{{\rm {a}}n}^{ - 1}} } \bigg )^{ - 1}}, \end{equation*}

where *Z*_a*n*_ is the surface impedance of each subunit and *N* is the number of subunits. Based on Eq. (5), a global optimization is conducted, aiming for an over-damped impedance (see Note 9 within the online supplementary material). From Fig. 3(b), it is evident that an intensive mode density is achieved over the full spectrum from 320 to 6400 Hz. Interestingly, it is found that denser resonance modes with larger half-maximum bandwidth are required for the modulation of low-frequency impedance, which may be explained by the more difficult modulation in the low-frequency regime. With the significant suppression of antiresonances resulting from intensive mode density, the acoustic impedance of the presented metamaterial is better modulated against dispersion than in previous work [30,31,49]. Both the theoretical and experimental acoustic resistances and reactances are very close to the optimization target [Fig. 3(c)], indicating the realization of over-damped resistance and broadband impedance matching over the working band. Accordingly, broadband quasi-perfect absorption is achieved theoretically and experimentally with a minimal thickness of 100 mm, manifesting an average value of 0.93 over the frequency band from 320 Hz to 6400 Hz [Fig. 3(d)]. The flat absorption spectrum provides an obvious validation of the suppression of antiresonance-induced absorption dips. Here, the slight difference between the theoretical and experimental results may be caused by the deviation in 3D printing, which has a precision of 0.1 mm. To intuitively illustrate the strong non-locality supported by the metamaterial, the near-field characteristics of the metamaterial are obtained by finite-element simulation (see Note 6 within the online supplementary material). The sound pressure and energy flux fields at two specific frequencies [labelled P and Q in Fig. 3(d)] are illustrated in Fig. 3(e). The simulation shows that at both frequencies, evident sound energy flux convolves around a number of subunits, revealing strong non-local coupling among them.

### Dual-function impedance modulation via a non-local metamaterial

**Figure 4.**
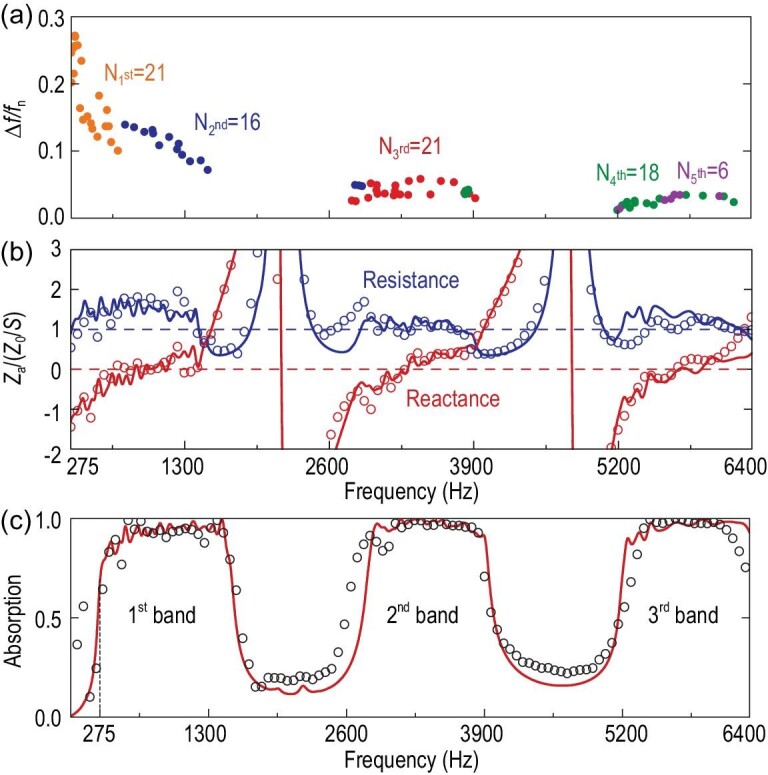
(a) Theoretical half-maximum bandwidths of resonance modes (1^st^ to 5^th^ orders). The modes with absorption peaks lower than 0.1 are ignored in the graph. (b) Theoretical (lines) and measured (circles) acoustic resistances and reactances of the multiband metamaterial absorber. (c) The corresponding theoretical (lines) and measured (circles) absorption spectra.

To further demonstrate the impedance modulating capability of our non-local metamaterial, we designed a dual-function metamaterial, which realizes frequency-selective impedance matching and a hard boundary. In the frequency bands with intensive modes, the induced strong non-local coupling enables the metamaterial to function as an absorbing boundary. In the frequency bands with sparse modes, the absence of resonance modes results in weak absorption, and the metamaterial serves as a reflective boundary. As shown in Fig. 4(a) and (b), the resonance modes are concentrated in three bands (275 Hz to 1517 Hz, 2797 Hz to 3943 Hz and 5200 Hz to 6400 Hz). Meanwhile, we maintain the over-damped status of the system by manipulating the acoustic resistance and reactance to be around 1.2 and 0, respectively. As a result, the dual-function metamaterial achieves quasi-perfect absorption in the three mode-concentrated bands and abruptly transfers into strong reflection in the mode-sparse bands. The thickness of the metamaterial is 100 mm, which also achieves the minimal thickness required by the causality principle. The theoretical impedance and absorption spectra are in good accordance with the experimental results [Fig. 4(c)]. The realization of the dual-function boundary shows great capability of the non-local metamaterial in impedance modulation.

## CONCLUSION

To summarize, we have investigated the underlying physics of optimal sound-absorbing systems and explored the effect of strong non-local coupling in broadband impedance modulation. The results reveal that over-damping is a general nature of optimal sound-absorbing materials. Furthermore, we demonstrated that strong non-local coupling can effectively suppress the dispersion due to antiresonances, leading to superior broadband impedance modulation. To demonstrate our concept, an over-damped impedance boundary is constructed via a non-local metamaterial. By employing 36 subunits, strong no-locality in the near field is generated and, meanwhile, the excessive response of the system is effectively reduced. Consequently, broadband quasi-perfect absorption without evident antiresonance-induced absorption dips is realized, reaching the minimal thickness required by the causality constraint. We also presented a dual-function design capable of frequency-selective absorption and reflection by redistributing the resonance modes. The concepts of the over-damped recipe and reduced excessive response recipe presented in this work may also contribute to impedance modulation in other wave systems. The methodology here should also have an impact on the realization of other artificial boundaries for radiation pattern control, wave manipulation in flow ducts, diffuse reflection and acoustic design for halls. Therefore, from a higher perspective, our work may inspire the exploration of extraordinary wave manipulation.

## Supplementary Material

nwab171_Supplemental_File
