# Supplementary material for: Broadband impedance modulation via non-local acoustic metamaterials
Source: Natl Sci Rev. 2021 Sep 11;9(8):nwab171. doi: 10.1093/nsr/nwab171 (PMC9440717; doi:10.1093/nsr/nwab171)
Supplement: nwab171_Supplemental_File [file nwab171_supplemental_file.pdf]

Supplemental Informations for “Broadband impedance modulation via non-local acoustic metamaterials”

Zhiling Zhou,[1,†] Sibo Huang,[1,2,†] Dongting Li,[1] Jie Zhu,[2,3,*] and Yong Li,[1,*]

[1] *Institute of Acoustics, School of Physics Science and Engineering, Tongji University, Shanghai 200092, China*

[2] *Department of Mechanical Engineering, The Hong Kong Polytechnic University, Hung Hom, Kowloon, Hong Kong, China*

[3] *The Hong Kong Polytechnic University Shenzhen Research Institute, Shenzhen 518057, China*

[*]Corresponding authors. E-mails: jie.zhu@polyu.edu.hk; yongli@tongji.edu.cn

[†]Equally contributed to this work.

Supplementary Note 1

Quality factor of Helmholtz resonator and the causality principle

The quality factor is widely used to describe the resonant characteristics of resonators. For a Helmholtz resonator, the relation between the relative bandwidth of the resonance peak and the quality factor Q satisfies [23]

$$\frac{1}{Q} = \frac{\Delta f}{f_\mathrm{r}} = \frac{(1+R_\mathrm{a})2\pi L}{\lambda_\mathrm{r}}, \tag{S1}$$

where Δf is the half-maximum relative bandwidth, f_r and λ_r are the resonance frequency and wavelength, R_a is the normalized acoustic resistance, and L denotes the thickness of HR. For perfect absorption when $R_\mathrm{a} = 1$, L can be solved and written as

$$L=\frac{1}{4\pi}\frac{\Delta f}{f_\mathrm{r}}\lambda_\mathrm{r}. \tag{S2}$$

Make $\Delta f = f_2 - f_1$, then

$$L = \frac{\lambda_\mathrm{r}^2}{4\pi}(\frac{1}{\lambda_2} - \frac{1}{\lambda_1}) = \frac{\lambda_\mathrm{r}^2}{4\pi}\left[\mathrm{F}(\lambda_2) - \mathrm{F}(\lambda_1)\right] = \frac{\lambda_\mathrm{r}^2}{4\pi}\int_{\lambda_2}^{\lambda_1}\frac{1}{\lambda^2}\mathrm{d}\lambda, \tag{S3}$$

where λ_1 and λ_2 are the corresponding wavelengths, and $\mathrm{F}(\lambda) = 1/\lambda$ is the primitive function. The two terms in parenthesis have a dimension of length, which reflects and quantifies the frequency response of the HR. The integral over the interval from λ_2 to λ_1 is obtained via the Newton-Leibniz formula. Obviously, Eq. (S3) possesses an analogous mathematical form

to Eq. (2) in the main text. This equation implies that to keep the same L, the interval $\Delta\lambda = (\lambda_2 - \lambda_1)$ decreases (increases) with λ_{r} increasing (decreasing), i.e. the bandwidth of the resonance peak is proportional to the resonance frequency. In this sense, the quality factor Q is equivalent to the causality principle. However, for broadband scenario, the absorption spectrum can not be quantified simply by a half-maximum bandwidth and thus the quality factor is invalid.

In addition, some conclusions can be drawn from the quantitative relation contained in the causality principle. First, for a certain thickness L, the absorption coefficient decreases (increases) with an increasing (decreasing) working bandwidth to keep the same integral value. Second, for a certain frequency band, the minimal thickness $L_{\min}$ increases (decreases) with an increasing (decreasing) absorption coefficient. However, the definition of optimality is not restricted by certain bandwidths or absorption coefficients. For instance, a material exhibiting low absorption or narrow bandwidth can still be optimal as long as the condition $L = L_{\min}$ is satisfied.

Supplementary Note 2

The response functions in Fig. 1

In Fig. 1b, we use auxiliary function as a mathematical fitting of the absorption spectrum. Generally, it can be summarized by a form of function expressed as $A = [\tan^{-1}(af + b) + \pi/2]/c + d$. By carefully reducing the coefficients a, b, and c, and choosing an appropriate intercept d, three functions increasing monotonically with descending derivative are obtained. The functions corresponding to the green, red, and blue curves are

$$\begin{aligned} A_1 &= [\tan^{-1}(0.15f - 65) + \pi/2]/(1.01\pi) - 0.0048, \\ A_2 &= [\tan^{-1}(0.03f - 12.4) + \pi/2]/(0.99\pi) - 0.025, \\ A_3 &= [\tan^{-1}(0.009f - 3.1) + \pi/2]/(0.91\pi) - 0.1092. \end{aligned} \tag{S4}$$

Supplementary Note 3

1. End correction of the embedded neck

At the port of the embedded neck, the vibration of the air column is influenced by the sound pressure field outside the neck, which causes a mass end correction of the neck. For a circular embedded neck in a square tube with a diameter d, if the dimension ratio $\xi < 0.4$,

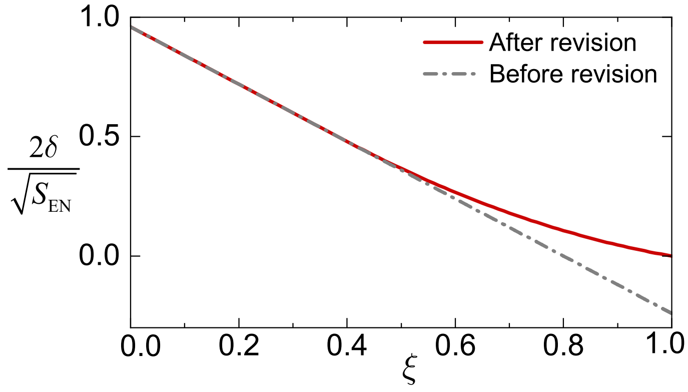

FIG. S1. Revised end correction of the CNEHR.

the end correction can be expressed as [42]

$$\delta = 0.48\sqrt{S_{\rm EN}}(1 - 1.25\xi), \tag{S5}$$

where $S_{\rm EN}$ is the cross-sectional area of the embedded neck, $\xi = d/\min(a, b)$, and a and b are the length and width of the square tube. As ξ approaches 1, the end correction decreases and converts to a small positive value, while Eq. (S5) gives a negative one. This violation would cause obvious deviation in high frequency regime. To amend this violation, we revise the end correction for $\xi \geq 0.4$ as

$$\delta = \sqrt{S_{\rm EN}}(\mathrm{a}\xi^2 + \mathrm{b}\xi + \mathrm{c})\Big/2, \tag{S6}$$

where a=2/3, b=-26/15, c=16/15 are the fitting coefficients. Fig. S1 shows that after the revision, the end correction agrees well with the result presented by Ingard [42] when $\xi \geq 0.4$.

2. Surface impedance analysis based on the transfer matrix method

As mentioned in the main text, the lumped-parameter impedance model is not valid for CNEHR in high frequency regime. Therefore, we developed the transfer matrix method to analyze the surface impedance of the CNEHR. Then, according to the acoustic continuity conditions (the continuity of sound pressure and volume velocity), the transfer matrix at different position inside the CNEHR can be deduced to relate the sound pressure and volume velocity at different positions (Fig. S2). This involves several equations expressed as

$$\begin{pmatrix} P_{\rm x_i} \\ V_{\rm x_i} \end{pmatrix} = \mathrm{M}_{\rm x_i} \begin{pmatrix} P_{\rm x_{i-1}} \\ V_{\rm x_{i-1}} \end{pmatrix}, \tag{S7}$$

when i takes different number (8, 7, 6, 5, 4, 3, 2), $\mathrm{M}_{\mathrm{x_i}}$, $P_{\mathrm{x_i}}$, $P_{\mathrm{x_{i-1}}}$, $V_{\mathrm{x_i}}$ and $V_{\mathrm{x_{i-1}}}$ respectively denotes the transfer matrix, sound pressure and volume velocity at x_8,x_7,x_6,x_5,x_4,x_3,x_2,x_1. Note that all the complex wave numbers and complex characteristic impedance in each part of the structure are calculated after considering the viscous and thermal dissipation in the acoustic boundary layer on the wall [43]. The following section gives the specific transfer matrix. The details of the deduction can be found in our previous work [26]. At the upper port of EN_1 (x_8), the end correction of the acoustic impedance can be added by employing the transfer matrix

$$\mathrm{M}_{\mathrm{EN_1,UP}}=\begin{pmatrix} 1 & \mathrm{j}k\left(\delta_1-\mathrm{j}\sqrt{2\eta/\omega\rho_0}\right)Z_0\Big/S_{\mathrm{EN_1}} \\ 0 & 1 \end{pmatrix}, \tag{S8}$$

where k and ω are the wave number and angular frequency of air in free space, $Z_0 = \rho_0 c_0$ and η are the characteristic impedance and dynamic viscosity of static air, δ_1 is the end correction of EN_1 relative to incident field, $kZ_0\sqrt{2\eta/\omega\rho_0} = \sqrt{2\eta\omega\rho_0}$ represents the surface resistance at the port of a circular tube, and $S_{\mathrm{EN_1}}$ is the cross-sectional area of EN_1. Inside the channel of EN_1 (x_7), the transfer matrix is

$$\mathrm{M}_{\mathrm{EN_1}}=\begin{pmatrix} \cos(k_{\mathrm{EN_1}}t_1) & \mathrm{j}\sin(k_{\mathrm{EN_1}}t_1)Z_{\mathrm{EN_1}}/S_{\mathrm{EN_1}} \\ \mathrm{j}\sin(k_{\mathrm{EN_1}}t_1)S_{\mathrm{EN_1}}/Z_{\mathrm{EN_1}} & \cos(k_{\mathrm{EN_1}}t_1) \end{pmatrix}, \tag{S9}$$

where $k_{\mathrm{EN_1}}$ and $Z_{\mathrm{EN_1}} = \rho_{\mathrm{EN_1}}c_{\mathrm{EN_1}}$ are the complex wave number and complex characteristic impedance of air inside EN_1. At the lower port of EN_1 (x_6), the region A around EN_1 splits the wave into two flows, where the boundary conditions can be expressed as $P_{x_7} = P_{x_6} = P_{\mathrm{A}}, V_{x_7} = V_{x_6} + V_{\mathrm{A}}$ and $P_{\mathrm{A}}/V_{\mathrm{A}} = -\mathrm{j}\cot[k_{\mathrm{A}}(t_1-b)]Z_{\mathrm{A}}/S_{\mathrm{A}}$. Considering the end correction of acoustic impedance and the boundary conditions, it can be deduced that $\begin{pmatrix} P_{x_7} \\ V_{x_7} \end{pmatrix} = \mathrm{M}_{\mathrm{EN_1,DW}}\begin{pmatrix} P_{x_6} \\ V_{x_6} \end{pmatrix}$, where

$$\mathrm{M}_{\mathrm{EN_1,DW}}=\begin{pmatrix} 1 & \mathrm{j}k\left(\delta_2-\mathrm{j}\sqrt{2\eta/\omega\rho_0}\right)Z_0\Big/S_{\mathrm{EN_1}} \\ 0 & 1 \end{pmatrix}\begin{pmatrix} 1 & 0 \\ \mathrm{j}\tan[k_{\mathrm{A}}(t_1-b)]S_{\mathrm{A}}/Z_{\mathrm{A}} & 1 \end{pmatrix}, \tag{S10}$$

where k_{A} and $Z_{\mathrm{A}} = \rho_{\mathrm{A}}c_{\mathrm{A}}$ are the complex wave number and complex characteristic impedance of air in region A, δ_2 is the end correction of EN_1 relative to the cavity, and

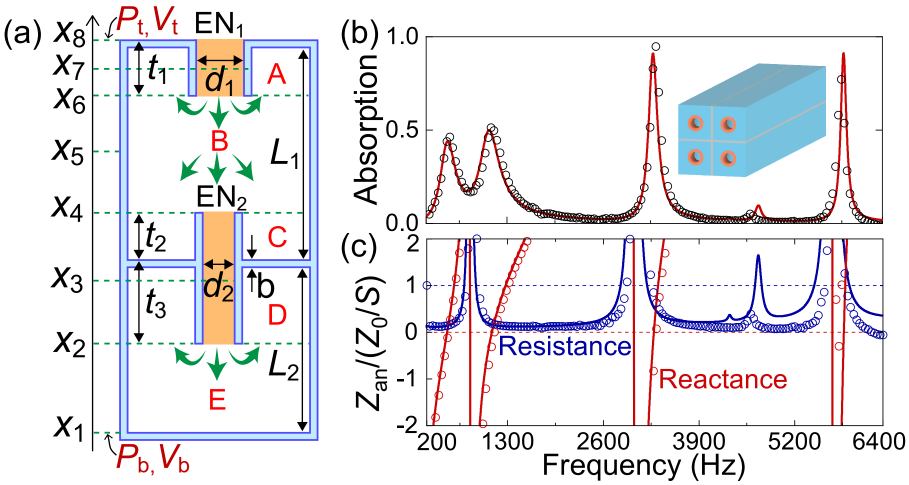

FIG. S2. Surface impedance analysis of the CNEHR. (a) Schematic of the CNEHR. (b) Theoretical (lines) and measured (circles) absorption spectra of the CNEHR subunit. (c) Theoretical (lines) and measured (circles) normal acoustic resistance and reactance of the CNEHR subunit.

$S_\mathrm{A} = wh - \pi(d_1/2 + b)^2$ is the cross-sectional area of region A. Inside the region B (x_5), the transfer matrix is

$$\mathrm{M_B}=\begin{pmatrix} \cos[k_\mathrm{B}(L_1 - t_1 - t_2 + b)] & \mathrm{j}\sin[k_\mathrm{B}(L_1 - t_1 - t_2 + b)]Z_\mathrm{B}/S_\mathrm{B} \\ \mathrm{j}\sin[k_\mathrm{B}(L_1 - t_1 - t_2 + b)]S_\mathrm{B}/Z_\mathrm{B} & \cos[k_\mathrm{B}(L_1 - t_1 - t_2 + b)] \end{pmatrix}, \tag{S11}$$

where k_B and $Z_\mathrm{B} = \rho_\mathrm{B}c_\mathrm{B}$ are the complex wave number and complex characteristic impedance of air inside region B, and $S_\mathrm{B} = wh$ is the cross-sectional area of region B. Similarly, the transfer matrix at x_4, inside EN$_2$ (x_3), at x_2, and inside region E (x_1) can be obtained as follows:

$$\mathrm{M_{EN_2,UP}}=\begin{pmatrix} 1 & 0 \\ \mathrm{j}\tan(k_\mathrm{C}t_2)S_\mathrm{C}/Z_\mathrm{C} & 1 \end{pmatrix}\begin{pmatrix} 1 & \mathrm{j}k\left(\delta_3 - \mathrm{j}\sqrt{2\eta/\omega\rho_0}\right)Z_0\Big/S_{\mathrm{EN}_2} \\ 0 & 1 \end{pmatrix}, \tag{S12}$$

where k_C and $Z_\mathrm{C} = \rho_\mathrm{C}c_\mathrm{C}$ are the complex wave number and complex characteristic impedance of air in region C, δ_3 is the end correction of EN$_2$ relative to the cavity, $S_\mathrm{C} = wh - \pi(d_2/2 + b)^2$ and S_{EN_2} are the cross-sectional areas of regions C and EN$_2$;

$$\mathrm{M_{EN_2}}=\begin{pmatrix} \cos[k_{\mathrm{EN}_2}(t_2+t_3)] & \mathrm{j}\sin[k_{\mathrm{EN}_2}(t_2+t_3)]Z_{\mathrm{EN}_2}/S_{\mathrm{EN}_2} \\ \mathrm{j}\sin[k_{\mathrm{EN}_2}(t_2+t_3)]S_{\mathrm{EN}_2}/Z_{\mathrm{EN}_2} & \cos[k_{\mathrm{EN}_2}(t_2+t_3)] \end{pmatrix}, \tag{S13}$$

where k_{EN_2} and $Z_{\mathrm{EN}_2} = \rho_{\mathrm{EN}_2}c_{\mathrm{EN}_2}$ are the complex wave number and complex characteristic impedance of air inside EN$_2$, and S_{EN_2} is the cross-sectional area of EN$_2$;

$$\mathrm{M_{EN_2,DW}}=\begin{pmatrix} 1 & \mathrm{j}k\left(\delta_3 - \mathrm{j}\sqrt{2\eta/\omega\rho_0}\right)Z_0\Big/S_{\mathrm{EN}_2} \\ 0 & 1 \end{pmatrix}\begin{pmatrix} 1 & 0 \\ \mathrm{j}\tan[k_\mathrm{D}(t_3 - b)]S_\mathrm{D}/Z_\mathrm{D} & 1 \end{pmatrix}, \tag{S14}$$

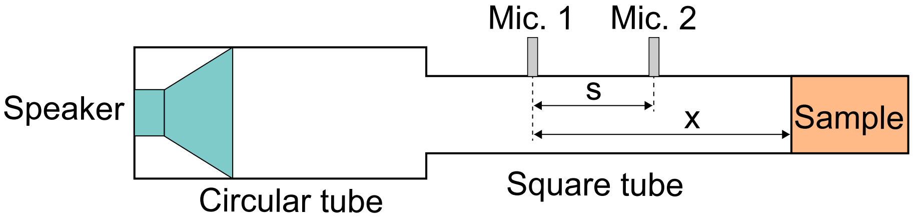

FIG. S3. Schematic of impedance tube measurement. The distances between Mic. 1 and Mic. 2 and the distance between Mic. 1 and the sample are denoted as s and x, respectively.

where k_D and $Z_\mathrm{D} = \rho_\mathrm{D} c_\mathrm{D}$ are the complex wave number and complex characteristic impedance of air in region D, and $S_\mathrm{D} = S_\mathrm{C}$ is the cross-sectional area of region C;

$$\mathrm{M_E}=\begin{pmatrix} \cos[k_\mathrm{E}(L_2-t_3+b)] & \mathrm{j}\sin[k_\mathrm{E}(L_2-t_3+b)]Z_\mathrm{E}/S_\mathrm{E} \\ \mathrm{j}\sin[k_\mathrm{E}(L_2-t_3+b)]S_\mathrm{E}/Z_\mathrm{E} & \cos[k_\mathrm{E}(L_2-t_3+b)] \end{pmatrix}, \tag{S15}$$

where k_E and $Z_\mathrm{E} = \rho_\mathrm{E} c_\mathrm{E}$ are the complex wave number and complex characteristic impedance of air in region E, and $S_\mathrm{E} = S_\mathrm{B}$ is the cross-sectional area of region E. Then the total transfer matrix T can be expressed as:

$$\mathrm{T} = \begin{pmatrix} \mathrm{T_1} & \mathrm{T_2} \\ \mathrm{T_3} & \mathrm{T_4} \end{pmatrix} = \mathrm{M_{EN_1,UP}} * \mathrm{M_{EN_1}} * \mathrm{M_{EN_1,DW}} * \mathrm{M_B} * \mathrm{M_{EN_2,UP}} * \mathrm{M_{EN_2}} * \mathrm{M_{EN_2,DW}} * \mathrm{M_E}. \tag{S16}$$

Combined with the boundary condition $V_\mathrm{b} = 0$ (hard boundary), the surface impedance of the CNEHR is written as $Z_\mathrm{an} = \mathrm{T_1}/T_3$.

To verify the acoustic impedance obtained above, we fabricated a 3D-printed sample of the CNEHR with the geometric parameters t_1=5 mm, t_2=20 mm, t_3=15 mm, d_1=3.9 mm, d_2=4.8 mm, w=10 mm, h=10 mm, L_1=60 mm, L_2=40 mm, and b=1.5 mm, and assembled a 2×2 array with it (Fig. S2b). It can be observed from Fig. S2b-c that, after considering the intrinsic loss of the square tube, the measured results agree well with the theory, which verifies the high accuracy of the surface impedance obtained from the transfer matrix method.

Supplementary Note 4

Transfer function method measurement and the revision for intrinsic loss of the impedance tube

The measurement is based on the transfer function method in accordance with ASTM C384-04(2011) and ASTM E1050-12. A circular impedance tube with a diameter of 100 mm

(Brüel & Kjær type-4206) connected by a square impedance tube with a cross-section area of 25×25 mm^2 was set up to generate and guide the sound wave. The sample was placed at the end of the square tube. The loud speaker installed at the port of the circular tube was powered by an amplifier (Brüel & Kjær type-2734) and generated a white noise signal that would eventually be reflected by the sample (Fig. S2). Then the transfer functions could be obtained with the signal detected by two 1/4-inch condenser microphones (Brüel & Kjær type-4187) fixed at specific positions on the top of the square tube. When the end of the square tube is acoustically rigid, the reflection coefficient r^{et} of the empty tube can be obtained as

$$r^{\mathrm{et}} = \frac{H_{12}^{\mathrm{et}} - H_{\mathrm{I}}}{H_{\mathrm{R}} - H_{12}^{\mathrm{et}}} \mathrm{e}^{2\mathrm{j}kx}, \tag{S17}$$

where $H_{12}^{\mathrm{et}} = p_2^{\mathrm{et}}/p_1^{\mathrm{et}}$ with p_2^{et} and p_1^{et} being the sound pressure detected by Mic. 1 and Mic. 2, $H_{\mathrm{I}} = \mathrm{e}^{-\mathrm{j}ks}$ and $H_{\mathrm{R}} = \mathrm{e}^{\mathrm{j}ks}$ are the transfer functions of the incident and reflected waves, and k is the wave number of air in free space. After considering the intrinsic loss of the square tube, the reflection coefficient should be rigorously 1:

$$1 = \frac{H_{12}^{\mathrm{et}} - H_{\mathrm{I}}^{\mathrm{c}}}{H_{\mathrm{R}}^{\mathrm{c}} - H_{12}^{\mathrm{et}}} \mathrm{e}^{2\mathrm{j}k'x}, \tag{S18}$$

where $H_{\mathrm{I}}^{\mathrm{c}} = \mathrm{e}^{-\mathrm{j}k's}$ and $H_{\mathrm{R}}^{\mathrm{c}} = \mathrm{e}^{\mathrm{j}k's}$ are the transfer functions of the incident and reflected waves after considering the loss, and k' is the complex wave number in the square tube. The complex wave number k' can be solved with Eq. (S17) and Eq. (S18), which yields the reflection coefficient r^{sp} of the tested sample:

$$r^{\mathrm{sp}} = \frac{H_{12}^{\mathrm{sp}} - H_{\mathrm{I}}^{\mathrm{c}}}{H_{\mathrm{R}}^{\mathrm{c}} - H_{12}^{\mathrm{sp}}} \mathrm{e}^{2\mathrm{j}k'x} \tag{S19}$$

where $H_{12}^{\mathrm{sp}} = p_2^{\mathrm{sp}}/p_1^{\mathrm{sp}}$ with p_2^{sp} and p_1^{sp} being the sound pressure detected by Mic. 1 and Mic. 2 when the tested sample is placed at the end of the square tube. Then the absorption coefficient and acoustic impedance of the sample can be obtained with $A = 1 - |r^{\mathrm{sp}}|^2$ and $Z_{\mathrm{a}} = (\rho_0 c_0/S + r^{\mathrm{sp}})/(\rho_0 c_0/S - r^{\mathrm{sp}})$.

Supplementary Note 5

Reduction of excessive response via increasing the number of subunits

Here, we will demonstrate that increasing the number of subunits can reduce the excessive response of the metamaterials. We investigate the absorption spectra of metamaterials

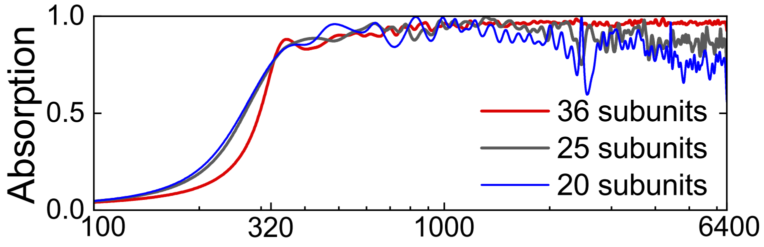

FIG. S4. Absorption spectra of metamaterials consisting of 20, 25 and 36 subunits with the same thickness (100 mm).

consisting of different numbers of subunits. For comparison, the absorption spectra of three metamaterials with 20, 25 and 36 subunits are shown in Fig. S4. According to the causality principle, the red, black and blue spectra respectively lead to minimal thicknesses of 97.3 mm, 97.3, and 96.7 mm, which are close to the actual thickness (100 mm). Therefore, all the structures reach the minimal thickness. However, different numbers of subunit are observed to yield distinct excessive responses. Actually, the number of subunit puts a strong constraint on the resonance characteristics of the metamaterial. Fewer subunits lead to bigger cross-sectional area for each subunit, which causes comparatively larger quality factor for each resonance mode. Generally, this signifies that the absorption peak contributed by each mode near 320 Hz is flatter. Hence, after coupling all the modes, the overall absorption spectra of the metamaterials with 20 and 25 subunits from 0 Hz to 320 Hz are inherently flatter than the spectrum of the one with 36 subunits, indicating larger excessive response and a bigger slope intuitively. Because the minimal thicknesses are almost the same, a higher absorption coefficient under 320 Hz inevitably leads to lower absorption above 320 Hz. Eventually, the absorption from 320 Hz to 6400 Hz is weakened and oscillates evidently. Therefore, to reduce the excessive response, we can employ a relatively larger number of subunits. It should be noted that when the thickness of the subunits' side walls is fixed, employing too many subunits will reduce the volume fraction and increase the minimal thickness or decrease the absorption efficiency. The number of subunits should be a suitably large number.

Excessive response is an intrinsic feature of the coupled-resonant system that can be weakened by altering the resonance modes to improve absorption performance in the operating spectrum. Especially in low frequency impedance-matching design, the excessive response plays a more important role in the absorption performance modulation.

Supplementary Note 6

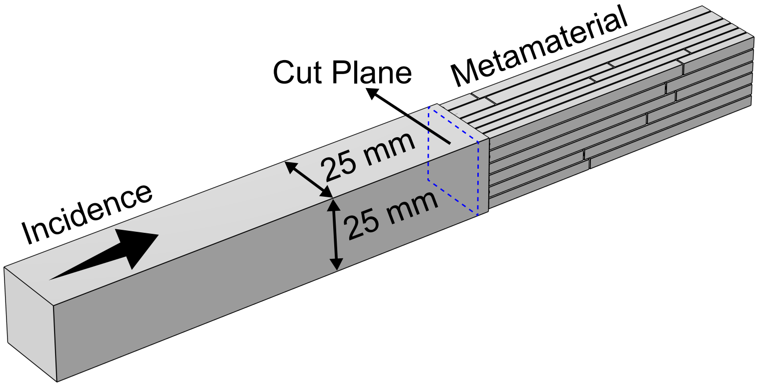

FIG. S5. Air domain of simulation model.

Simulation details

In Fig. 3e of the main text, commercial software COMSOL Multiphysics with the preset Pressure Acoustics Frequency Domain module is employed to perform finite-element simulation. The geometric model of the metamaterial is built according to the parameters listed in Supplementary Note 7. The surrounding medium is air with a density $\rho_0 = 1.21\text{kg/m}^3$ and sound speed $c_0 = 343\text{m/s}$. All of the structures' boundaries are set as "Sound Hard Boundary" due to the huge impedance mismatch between air and the resin walls. Besides, the inner surfaces of the structures are set as "Thermoviscous Boundary Layer Impedance Interface" to calculate the thermoviscous effect. An incident plane wave guided by a square duct normally impinges on the metamaterial (Fig. S5). As a consequence, the pressure and energy flux field in Fig. 3e are obtained by setting a Cut Plane 0.1 mm above the metamaterial. The energy flux is defined as $I_{x(y)} = \frac{1}{2}\text{Re}(p^* v_{x(y)})$, where p is the sound pressure, $v_{x(y)}$ is the particle velocity with respect to the $x(y)$ direction, and $I_{x(y)}$ is the energy flux with respect to the $x(y)$ direction.

Supplementary Note 7

Influence of the arrangement of subunits

To explore the influence caused by the arrangement of subunits, we simulated the absorption spectra of two sets of comparative structures (Fig. S6). The first structure was composed of 2 subunits. We changed the arrangement in terms of the distance between the two subunits to evaluate the effect of distance on absorption. The second structure was composed of 12 subunits. We changed the arrangement in terms of the order of subunits to evaluate the effect of multi-subunits' order on absorption. The resulting absorption spectra

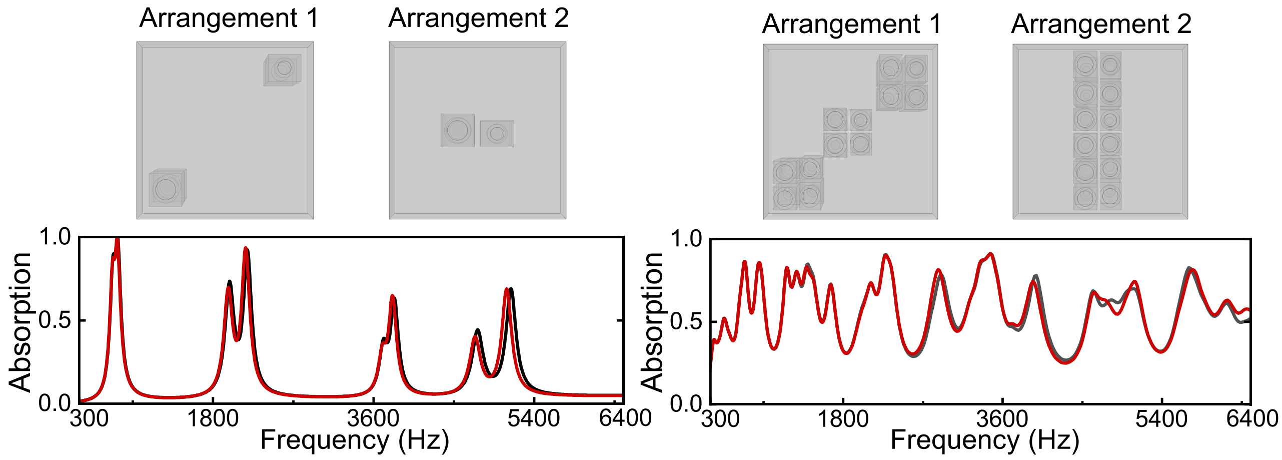

FIG. S6. Simulated absorption spectra of two structures composed of 2 subunits and 12 subunits with different arrangements. The black and red curves represent the absorption spectra of arrangement 1 and 2. The parameters of the 2-subunit structure are listed in Supplementary Note 11 Table III. The parameters of the 12-subunit structure are those listed in Supplementary Note 11 Table **??** unit 13-24.

in both cases have small deviation after the change in subunit order, which verifies that subunit arrangement is not the primary influence factor in the non-local coupling and can be ignored for our structures in the interest of frequency. Additionally, we note that although the coupling among the subunits is not sensitive to their arrangement in our design, the coupling is greatly affected by other important factors such as the resonance frequency, mode density and acoustic resistance.

Supplementary Note 8

Resonance mode of each subunit of the metamaterials in the main text

To further demonstrate the near-continuous resonance mode of the metamaterial in Fig. 3 and Fig. 4 in the main text, we calculate the absorption spectra of all the subunits using

$$A_{\mathrm{n}} = 1 - \left| \frac{Z_{\mathrm{an}} - Z_0/S}{Z_{\mathrm{an}} + Z_0/S} \right|^2, \tag{S20}$$

where A_{n} is the absorption coefficient of the n-th subunit. The overall absorption spectra of the metamaterial and the individual CNEHRs are shown in (Fig. S7). The numerous absorption peaks demonstrate the near-continuous resonance mode of the metamaterial.

Supplementary Note 9

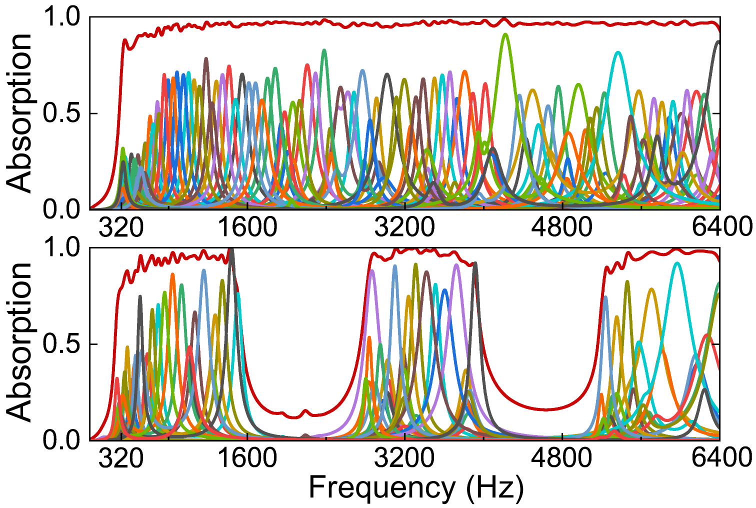

FIG. S7. Resonance mode of each subunit of the metamaterials in Fig. 3 and Fig. 4. The colored lines represent the absorption spectrum of individual CNEHR.

Design strategy for the non-local metamaterials

In our work, we appropriately harnessed the nonlocality in the near field to suppress the impedance oscillation at anti-resonance frequency. However, with the bandwidth broadening, more subunits are required to ensure broadband modulation of the impedance. Meanwhile, the coupling becomes more complicated. Thus, we employed optimization algorithm (genetic algorithm) to accomplish the non-local metamaterials in the main text. In the optimization, specific resistance and reactance values are expected in the target frequency band. As stated in the main text, the impedance matching condition requires the resistance to be 1 and the reactance to be 0. However, the inevitable small oscillation of the impedance will easily results in a resistance smaller than 1 in some frequency bands. Hence, to ensure the over-damped condition, the objective of resistance is larger than 1 in this study. The objective with respect to resistance and reactance is written as

$$\text{Objective} = \sum_{\mathrm{i}=1}^{\mathrm{N}} \left[|r_{\mathrm{a}}(f_{\mathrm{i}}) - 1.4| + |x_{\mathrm{a}}(f_{\mathrm{i}}) - 0| \right], \tag{S21}$$

where f_{i} is the chosen frequencies in the target band, and $r_{\mathrm{a}}(f_{\mathrm{i}})$ and $x_{\mathrm{a}}(f_{\mathrm{i}})$ is the normalized resistance and reactance at f_{i}. During the iteration, the objective gradually decreases and reaches a minimum where the optimized parameters of the device are obtained. The obtained results lead to the optimal absorption performance under our concept-based design, i.e., the given type and number of subunits. Because our results demonstrate that the relatively more intensive mode density can better suppress the dispersion of impedance and a smaller volume

fraction of the material (ϕ) allows a thinner structure, we think it is possible to improve the structure by further increasing the mode density and decreasing the volume fraction. Note that in our design, keeping the same length L for all the CNEHRs ensures that the metamaterial possesses a bigger effective volume fraction, which enables the metamaterial to approach the minimal thickness governed by the causality principle.

Supplementary Note 10

Effect of non-local coupling on impedance manipulation

The coupled mode theory is provided to describe the effect of non-local coupling on impedance matching. For simplicity but without loss of generality, we investigate a system coupled with three modes, where the mode amplitudes are expressed as

$$\frac{d}{dt}\tilde{a}_1 = (j\omega_1 - \gamma_1 - \Gamma_1)\tilde{a}_1 + j\sqrt{\gamma_1}\tilde{S}_i^+ + j\sqrt{\gamma_1}(j\sqrt{\gamma_2}\tilde{a}_2 + j\sqrt{\gamma_3}\tilde{a}_3), \tag{S22}$$

$$\frac{d}{dt}\tilde{a}_2 = (j\omega_2 - \gamma_2 - \Gamma_2)\tilde{a}_2 + j\sqrt{\gamma_2}\tilde{S}_i^+ + j\sqrt{\gamma_2}(j\sqrt{\gamma_1}\tilde{a}_1 + j\sqrt{\gamma_3}\tilde{a}_3), \tag{S23}$$

$$\frac{d}{dt}\tilde{a}_3 = (j\omega_3 - \gamma_3 - \Gamma_3)\tilde{a}_3 + j\sqrt{\gamma_3}\tilde{S}_i^+ + j\sqrt{\gamma_3}(j\sqrt{\gamma_1}\tilde{a}_1 + j\sqrt{\gamma_2}\tilde{a}_2), \tag{S24}$$

where S_i^+ represents the incident waves, $\omega_{1(2,3)}$ is the resonance frequency of mode 1(2,3), $\gamma_{1(2,3)}$ is the corresponding radiative decay rate, and $\Gamma_{1(2,3)}$ represents the dissipative decay rate associated with the intrinsic loss. The third group of terms in Eqs. S22-S24 represents interactions among the modes, i.e., the non-local coupling effect. In the absence of these coupling terms, the resonances become local and we denote the local resonances as $a_{1,0}$, $a_{2,0}$, and $a_{3,0}$.

Figures. S8-S10 illustrate the influences of non-local coupling with increasing mode density, where we can find that the real and imaginary parts of the resonances' amplitudes are altered due to the interactions among the resonances. Here, the individual non-local factor (F_{ni}) and the overall non-local factor (F_{no}) are introduced to evaluate the influence of the non-local coupling on the individual modes and the overall system, respectively, which are expressed as

$$F_{\mathrm{ni}i} = \frac{|a_i - a_{i,0}|}{a_{i,0}}, \tag{S25}$$

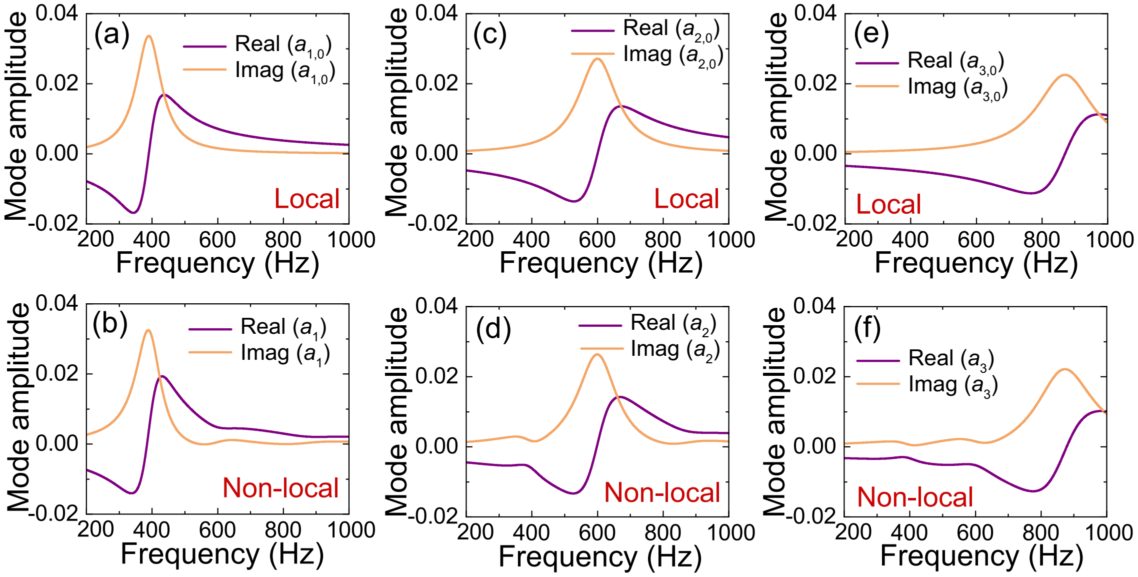

FIG. S8. Mode amplitude of the local and non-local acoustic systems under the relatively sparse mode density. $\omega_1/(2\pi)$, $\omega_2/(2\pi)$ and $\omega_3/(2\pi)$ are 390 Hz, 600 Hz and 870 Hz, respectively. γ_1, γ_2, and γ_3 are $0.04\omega_1$, $0.04\omega_2$, $0.04\omega_3$, respectively. Γ_1, Γ_2 and Γ_3 are $0.08\omega_1$, $0.08\omega_2$, $0.08\omega_3$, respectively.

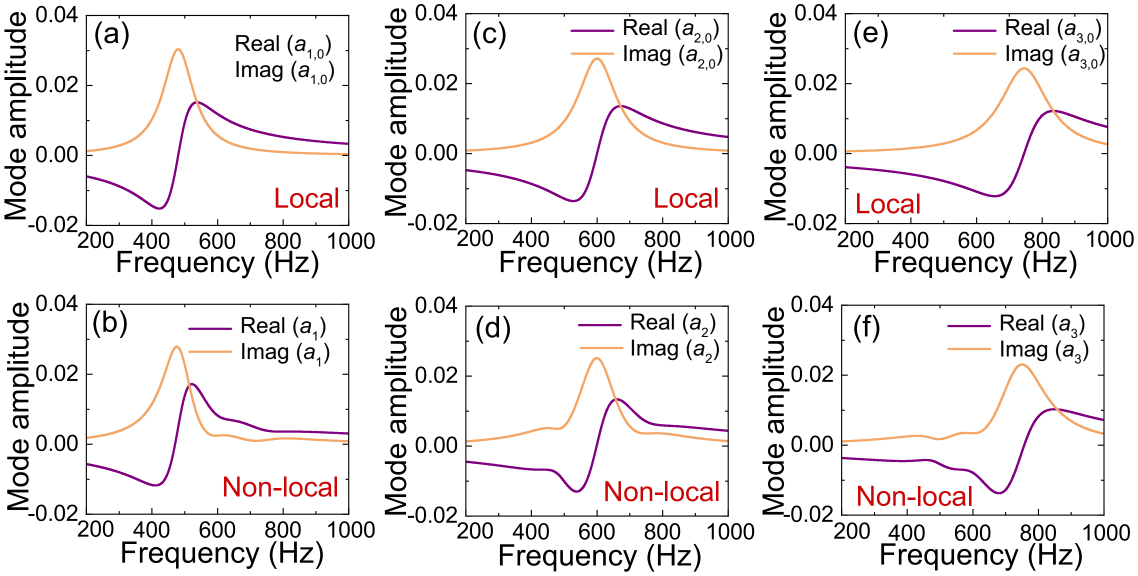

FIG. S9. Mode amplitude of the local and non-local acoustic systems under the relatively moderate mode density. $\omega_1/(2\pi)$, $\omega_2/(2\pi)$ and $\omega_3/(2\pi)$ are 480 Hz, 600 Hz and 745 Hz, respectively. γ_1, γ_2, and γ_3 are $0.04\omega_1$, $0.04\omega_2$, $0.04\omega_3$, respectively. Γ_1, Γ_2 and Γ_3 are $0.08\omega_1$, $0.08\omega_2$, $0.08\omega_3$, respectively.

$$F_{\mathrm{no}} = \sum_{i=1}^{N} F_{\mathrm{n}ii}, \tag{S26}$$

where we can substitute $i = 1, 2, 3$ and $N = 3$ to analyze the three-state system presented

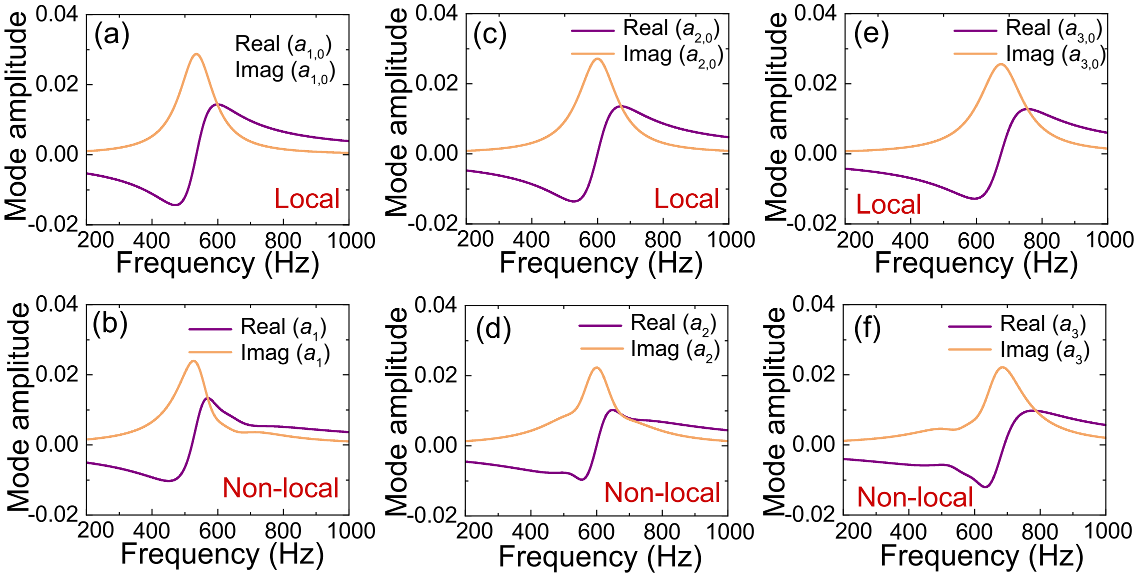

FIG. S10. Mode amplitude of the local and non-local acoustic systems under the relatively intensive mode density. $\omega_1/(2\pi)$, $\omega_2/(2\pi)$ and $\omega_3/(2\pi)$ are 535 Hz, 600 Hz and 675 Hz, respectively. γ_1, γ_2, and γ_3 are $0.04\omega_1$, $0.04\omega_2$, $0.04\omega_3$, respectively. Γ_1, Γ_2 and Γ_3 are $0.08\omega_1$, $0.08\omega_2$, $0.08\omega_3$, respectively.

above. Further, the reflection coefficient of the coupled system, r, is given as [46]

$$r = 1 + 2i(\sqrt{\gamma_A}\frac{a_A}{S_i^+} + \sqrt{\gamma_B}\frac{a_B}{S_i^+}\sqrt{\gamma_C}\frac{a_C}{S_i^+}). \tag{S27}$$

Accordingly, the acoustic impedance (Z) of the system can be written as

$$Z = \frac{1+r}{1-r}. \tag{S28}$$

As shown in Fig. S11, it can be observed that with increasing mode density, the individual non-local factors of mode 1 ($F_{\rm ni1}$), mode 2 ($F_{\rm ni2}$) and mode 3 ($F_{\rm ni3}$) and the overall non-local factor ($F_{\rm no}$) of the corresponding systems are enhanced. These results indicate that the relatively more intensive mode density can support stronger effect of non-local coupling on each mode. Furthermore, it can be found evidently from Figs. S11(b)-(c) that the dispersion of impedance occurs at the frequencies with the small $F_{\rm no}$, which will reduce the efficiency of impedance modulation and result in absorption dips for sound-absorbing metamaterials. However, this problem can be well solved when we construct the system with denser modes to support strong non-local coupling. In this manner, the dispersion of impedance can be suppressed, leading to improved modulation for impedance matching.

Supplementary Note 11

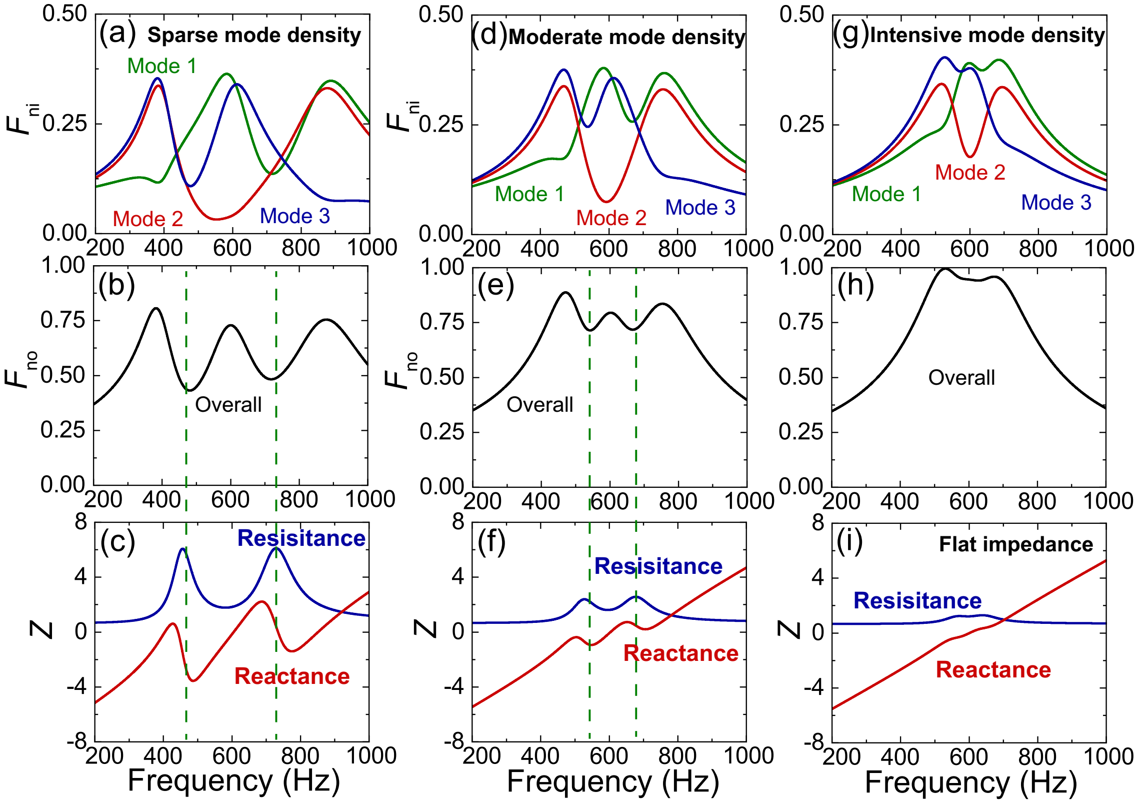

FIG. S11. (a)-(c) The individual non-local factor, overall non-local factor, and acoustic impedance of the system with relatively sparse mode density. (d)-(f) The individual non-local factor, overall non-local factor, and acoustic impedance of the system with relatively moderate mode density. (g)-(i) The individual non-local factor, overall non-local factor and acoustic impedance of the system with relatively intensive mode density.

Geometric parameters of several structures in the main text

To support our theory, several typical CNEHRs were designed to demonstrate the over-damped nature of optimal sound-absorbing system. In addition, we presented two non-local metamaterials to realize broadband absorption. The wall thicknesses of the front, middle, back panel (b_2) and the embedded neck (b_1) of the metamaterials are set as different values to guarantee maximal use of the space as well as sufficiently strong stiffness. The 3D printer can technically reach a precision of 0.1 mm, which is at the same level as that of the parameters of the presented acoustic metamaterials. The resonance frequency may slightly shift from the theoretical value due to fabrication imperfections, but it would not significantly affect the overall impedance modulation owing to the intensive mode density. The geometric parameters are listed in the following tables.

TABLE I. Geometry parameters (unit: mm) of the CNEHR corresponding to Fig. 2b-c.

**Unit No.**	t_1	t_2	t_3	d_1	d_2	w	h	L_1	L_2	b_1	b_2
1	-	1.0	-	2.1	2.1	13.0	13.0	50.0	50.0	1.0	1.0

TABLE II. Geometry parameters (unit: mm) of the CNEHRs corresponding to Fig. 2d-e. Note that since the acoustic impedance of the subunits in the metamaterials will be enlarged due to the change of surface area, their neck lengths t_1 are relatively shorter than the single structure presented here.

**Unit No.**	t_1	t_2	t_3	d_1	d_2	w	h	L_1	L_2	b_1	b_2
1	1.0	5.0	1.0	2.5	2.5	13.0	13.0	50.0	50.0	1.0	1.0
2	1.6	1.0	4.4	1.0	1.0	13.0	13.0	10.0	10.0	1.0	1.0

TABLE III. Geometry parameters (unit: mm) of the CNEHRs corresponding to Fig. S6.

**Unit No.**	t_1	t_2	t_3	d_1	d_2	w	h	L_1	L_2	b_1	b_2
1	1.0	1.0	1.0	3.0	3.0	5.0	5.0	50.0	50.0	1.0	1.0
2	1.0	1.0	1.0	2.0	2.0	5.0	4.0	50.0	50.0	1.0	1.0
